# Physicochemical characterization and 16S rRNA analysis of a direct-fed microbial from calf ruminal fluid and its protective effect on Sprague–Dawley rat gut barrier function

**DOI:** 10.1093/tas/txaf003

**Published:** 2025-01-09

**Authors:** Haiku D J Gómez-Velázquez, Laura González-Dávalos, Erika A de los Ríos, Juan de Dios Figueroa-Cárdenas, Alma Vázquez-Durán, Abraham Méndez-Albores, Armando Shimada, Ofelia Mora

**Affiliations:** Laboratorio de Rumiología y Metabolismo Nutricional (RuMeN), Facultad de Estudios Superiores-Cuautitlán (FESC), Universidad Nacional Autónoma de México (UNAM), Querétaro, Querétaro, Mexico; Laboratorio de Rumiología y Metabolismo Nutricional (RuMeN), Facultad de Estudios Superiores-Cuautitlán (FESC), Universidad Nacional Autónoma de México (UNAM), Querétaro, Querétaro, Mexico; Unidad de Microscopía, Instituto de Neurobiología, UNAM, Querétaro, Querétaro, México; Materiales Bio-orgánicos, CINVESTAV-Unidad Querétaro, Libramiento Norponiente No. 2000, Fraccionamiento Real de Juriquilla, Querétaro, Querétaro, México; Ciencia y Tecnología de Materiales, Unidad de Investigación Multidisciplinaria L14-A1 (Ciencia y Tecnología de Materiales), FESC, UNAM, Cuautitlán Izcalli, Estado de México, México; Ciencia y Tecnología de Materiales, Unidad de Investigación Multidisciplinaria L14-A1 (Ciencia y Tecnología de Materiales), FESC, UNAM, Cuautitlán Izcalli, Estado de México, México; Laboratorio de Rumiología y Metabolismo Nutricional (RuMeN), Facultad de Estudios Superiores-Cuautitlán (FESC), Universidad Nacional Autónoma de México (UNAM), Querétaro, Querétaro, Mexico; Laboratorio de Rumiología y Metabolismo Nutricional (RuMeN), Facultad de Estudios Superiores-Cuautitlán (FESC), Universidad Nacional Autónoma de México (UNAM), Querétaro, Querétaro, Mexico

**Keywords:** gut permeability, microbiota, probiotics, surface properties

## Abstract

This study aimed to characterize the physicochemical properties and microbiota composition of a direct-fed microbial (DFM) and evaluate its protective effect on intestinal permeability in Sprague–Dawley rats using fluorescein isothiocyanate dextran (FITC-d) as a biomarker. The DFM was further characterized using Fourier-transform infrared spectroscopy (FTIR), dynamic light scattering (DLS), environmental scanning electron microscopy (ESEM), energy-dispersive X-ray spectroscopy (EDS), and cell surface hydrophobicity (microbial adhesion to hexadecane [MATH] assay). The 16S rRNA gene was sequenced using short-read sequencing. In general, the DFM exhibited the characteristic FTIR bands associated with probiotic cells with a protein/carbohydrate ratio of 1.3:1. It was also found from the DLS analysis that the average particle size and surface electrical potential of the probiotic cells were 1,062 ± 77 nm and −32.6 ± 3.7 mV, respectively. ESEM studies confirmed the size of the cells (1,010 to 1,060 nm), showing a quasi-spherical cocci-type morphology, whereas EDS spectroscopy revealed a higher Nitrogen/Carbone ratio on the cell surface. Moreover, the MATH assay showed the hydrophobic character of the DFM (92% adhesion). Furthermore, based on the 16S rRNA gene analysis, the predominant genus in the DFM was *Streptococcus* (99%). Regarding the protective effect on the gut barrier, animals supplemented with 10^11^ CFU/mL exhibited a significantly reduced intestinal permeability compared with the control group. DFM supplementation also increased villi and crypt dimensions and Goblet cells (*P *< 0.05) in the ileum and cecum. These results demonstrate that the DFM presented adequate surface and colloidal properties that help maintain the functionality of the gut barrier.

## Teaser

The beneficial effects of a direct-fed microbial (DFM) isolated from calf rumen fluid were evaluated and found to enhance the intestinal barrier by reducing the absorption of the marker fluorescein isothiocyanate dextran. Additionally, physicochemical techniques, including Fourier-transform infrared spectroscopy, as well as size distribution and zeta-potential analysis were employed as low-cost and rapid alternatives to standard methods, providing preliminary support for effectively characterizing the DFM.

## Graphical Abstract

**Graphical Abstract:**
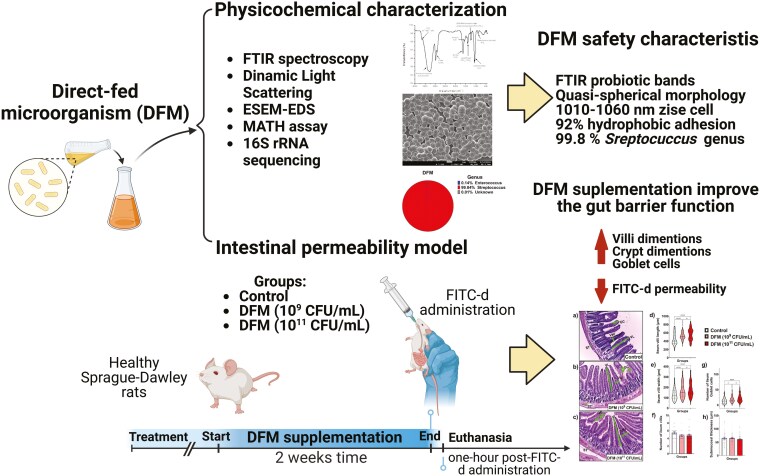

## Introduction

The intestinal barrier, a crucial element in maintaining health, is responsible for separating the external environment from the host environment. Its integrity, determined by the epithelium and the tight junction that seals the paracellular space, is a key factor (Gou et al., 2022). The properties of the selective and permeable intestinal tight junction can be modified in response to luminal nutrients or pathogens (Di Vincenzo et al., 2023). The intestinal gut microbiota plays a significant role in maintaining these functions correctly, sustaining homeostasis, and promoting health (Olvera-Rosales et al., 2021). Enteric dysbiosis, which compromises intestinal barrier integrity, is commonly associated with severe diseases, including diarrhea, inflammatory bowel disease, and irritable bowel syndrome (Mennigen and Bruewer, 2009; Haidmayer et al., 2020; Di Vincenzo et al., 2023). However, it can be ameliorated by dietary measures, stool transplantation, or the intake of direct-fed microbial (DFM) strains as dietary supplementation (Haidmayer et al., 2020; Jha et al., 2020; Reuben et al., 2022).

In ruminants, dietary supplementation of DFM is related to health benefits, as it may upregulate gene expression and protein synthesis involved in tight junction signaling, regulating the proliferation of intestinal epithelial cells to restore the intestinal mechanical barrier (Reuben et al., 2022). Several DFM treatments enhance intestinal health by stimulating the balance of gut microbiota, preventing the colonization of pathogens, secreting antimicrobial substances, increasing digestive capacity, and improving mucosal immunity (Dick et al., 2013; Abd El-Tawab et al., 2016; Reuben et al., 2022). However, the DFM has different modes of action and does not represent a single type of intervention to improve host animal health (Alawneh et al., 2020).

In addition, it has been demonstrated that DFM supplementation improves feed efficiency and weight gain in animals (Abd El-Tawab et al., 2016), including multispecies and multistrains supplementation such as yeast cultures, *Saccharomyces cerevisiae*, *Lactobacillus* spp. *L. rhamnoses*, *L. acidophilus, Bifidobacterium* spp., *Bacillus* spp., *Enterococcus faecium*, and *Enterococcus* spp., therefore, supplementation of multispecies and multistrain DFM typically results in improved beneficial effects on the host due to their different effects (Cangiano et al. 2020).

The microorganism population of DFM should be well-identified and comprehensively characterized to confirm their potential beneficial effects and safety. This is crucial to ensure that they meet the criteria to be included in the concept of “Generally Recognized as Safe” (GRAS) (FAO/WHO, 2001). These microorganisms are traditionally identified and cultivated using different nutritive culture mediums based on morphology and biochemical tests, and nowadays, the gold standard is through molecular biology methods, such as the 16S rRNA gene sequencing (Adhikari and Kwon, 2017; Shokryazdan et al., 2017). Although these methods are particular in their application, they are expensive, time-consuming, and require specialized and sophisticated training (Vinderola et al., 2019).

Thus, alternative methods that meet simplicity, reliability, high specificity, fast, and relatively inexpensive criteria should be considered an essential aspect of the DFM application in the livestock industry (Dziuba et al., 2007). For instance, Fourier-transform infrared spectroscopy (FTIR) and environmental scanning electronic microscopy (ESEM) techniques could help characterize some physicochemical properties of the microbial population (Deidda et al., 2022). FTIR is used to identify the functional groups present in the DFM population related to the vibration modes of these molecules by measuring infrared absorption spectra. Meanwhile, ESEM provides detailed images of the morphology and size of DFM in its natural state, with minor sample preparation.

In this context, various studies have demonstrated the effectiveness of the FTIR technique to identify and differentiate bacterial strains (Wenning et al., 2010; Deidda et al., 2022). However, most of these investigations are conducted on a particular strain genus, especially those related to the food industry (Wenning et al., 2010; Dziuba and Nalepa, 2012; Deidda et al., 2022). Until today, few studies point to their application in DFM characterization for the livestock industry.

In previous works (Rodríguez-González et al., 2022a, 2022b), we isolated and characterized a potential DFM candidate, A1D42 from the ruminal fluid of calves, with an exceptional in vitro mucus adhesion, sensitivity to antibiotics, and antagonist effect against other photogenic bacteria. Consequently, this study aimed to 1) characterize the physicochemical properties of the DFM (A1D42), 2) conduct a molecular characterization of the microbial species using 16S rRNA, and 3) evaluate its potential protective effect on gut permeability in Sprague–Dawley rats. This study represents a significant step toward identifying the benefits and drawbacks of DFM supplementation in gut health for subsequent ruminant experiments.

## Material and Methods

### Bacterial Consortia and Culture Conditions

The bacterial consortia were isolated from the ruminal fluid of 42-d-old clinically healthy F1 Holstein × Zebu calves (*n* = 3), as was reported in our previous work by Rodríguez-González et al. (2022a). The calves were raised at the Centro de Enseñanza, Investigación y Extensión en Ganadería Tropical (CEIEGT) of the Universidad Nacional Autónoma de México (FMVZ‐UNAM) in Veracruz, México. At birth, the calves were fed colostrum with their mothers until weaning. After this period, the calves were separated from the cow and moved to the rearing area, where they were fed a milk substitute (22% protein and 12% fat) and roughage ad libitum in the first week. They began to eat forage after the first week of age. All the animals were humanely sacrificed at CEIEGT by national and international regulations and ethical guidelines for animal welfare. The protocol was ethically approved by the Internal Committee for the Care and Use of Experimental Animals (CICUAE.DC-2019/4-2, UNAM). Immediately after the sacrifice, an incision was made in the abdominal cavity to expose the rumen, and rumen fluid samples were collected using a sterile syringe directly from the rumen to ensure sample purity and avoid contamination. The selection and identification of the potential DFM candidate were previously evaluated in our laboratory, as reported by Rodríguez-González et al. (2022a). To ensure reproducibility, aliquots of A1D42 consortia were placed into 80% glycerol and stored at −70 °C until further analysis. This A1D42 was the DFM used in this study.

The consortia were inoculated into 50 mL of De Man, Rogosa, and Sharpe (MRS, BD, Sparks, MD, USA) broth in anaerobic conditions at 37 °C for 18 h (Rodríguez-González et al., 2022a). The activated culture was inoculated into a my-control bioreactor (Applikon Biotechnology, Delft, NLD) containing 2.4 L of MRS broth for 18 h, reaching approximately 10^9^ CFU/mL to obtain fresh DFM. Bacterial viability was measured using the biomass probe sensor (Futura, ABE Trusted Technology, Delft, NLD) in the bioreactor system (Rodríguez-González et al., 2022b). Finally, the culture was concentrated by centrifugation (4000 g, 10 min), and the supernatant was discarded to obtain 10^9^ and 10^11^ CFU/mL doses. These doses were corroborated using the plate cultivation method. The samples were used fresh and hermetically stored in a Falcon tube protected from light and kept in anaerobic conditions. They were immediately used for physicochemical characterization or to obtain the doses for the in vivo experiment.

### Physicochemical Characterization

#### Fourier-transform infrared spectroscopy.

Information regarding the functional groups on the surface of the DFM was determined by Fourier-transform infrared spectroscopy (FTIR) spectral analysis according to the recommendations of Vázquez-Durán et al. (2021). Briefly, the spectrum of the concentrated bacterial cells was acquired in a Frontier SP8000 NIR/MIR spectrophotometer (Perkin Elmer, Waltham, MA, USA) accessorized with an in-compartment diamond ATR accessory (DuraSamplIR II, Smiths Detection, Warrington, UK). Thirty-two sequential scans in the 4,000 to 400 cm^−1^ range were collected with a resolution of 4 cm^−1^. The FTIR spectrum of the DFM includes not only the contributions of the concentrated bacterial cells but also a large contribution of the culture media; therefore, a spectrum of pure culture media (sterilized MRS broth) was also measured under identical conditions and subtracted from the spectrum of the DFM (subtraction factor = 0.827) using the Spectrum 10.4.2 software. A semi-quantitation analysis of 2 spectral regions (protein and carbohydrate) was performed by integrating the corresponding bands using the Origin 8 software (Microcal Software Inc., Northampton, MA, USA).

#### Hydrodynamic diameter, polydispersity index, and zeta-potential determinations.

The hydrodynamic diameter, polydispersity index (PDI), and zeta-potential measurements were acquired using a particle size analyzer (ZetaSizer Pro, Malvern Instruments, Worcestershire, UK). Samples of the concentrated bacterial cells were appropriately diluted with deionized water and adjusted at pH 6 to emulate the conditions of the intestinal environment. Subsequently, triplicates of each sample were analyzed in a disposable capillary cell (DTS1070) at 25 °C with an equilibration period of 2 min. Each measurement comprised 20 runs to ensure a stable reading, and results were meticulously analyzed using the ZS Xplorer software.

#### Bacterial cell surface hydrophobicity.

The hydrophobic surface character of the DFM was evaluated by means of the microbial adhesion to hexadecane (MATH) assay based on the recommendations of Rosenberg et al. (1980). Briefly, the concentrated bacterial cells (10^9^ CFU/mL) were suspended in 10 mmol KH_2_PO_4_ until absorption at 600 nm reached approximately 0.8 A. U by triplicate. The pH of the suspension was adjusted to 3 by adding HCl. Subsequently, triplicates of the suspension were mixed with an equal volume of hexadecane, and the mixture was vortexed for 1 min and allowed to stand for 20 min until complete phase separation. The absorbance of the aqueous phase was measured at 600 nm using a Cary 8,454 UV–Vis Diode Array System spectrophotometer (Agilent Technologies, Santa Clara, CA, USA). The percentage of microbial adhesion was computed using the following mathematical expression:


$$\begin{array}{l} Adhesion=\left(\frac{1-A_{1}}{A_{0}}\right) \end{array}$$

where $A_{0}$ and $\;A_{1}$are the initial and final absorbances of the cell suspension.

#### Environmental scanning electron microscopy (ESEM) and energy-dispersive x-ray spectroscopy (EDS) analyses.

The morphological characteristics and the multi-elemental analysis of the concentrated bacterial cells were investigated using an environmental scanning electron microscope accessorized with energy-dispersive X-ray fluorescence spectroscopy (Phillips XL30/40 ESEM-EDS, Eindhoven, NLD). Microscopy analysis was performed at 5,000, 7,500, and 15,000 × with an accelerating voltage of 2 kV. The multi-elemental analysis was performed using a high-performance XTrace microspot X-ray source, and the generated X-ray fluorescence spectrum was collected with the attached XFlash 6/10 silicon drift detector (Bruker Nano GmbH, Berlin, Germany). Micrographs were analyzed using the software ImageJ64 according to the recommendation of Schindelin et al. (2012).

### Extraction of Genomic DNA

Genomic DNA was extracted using the RBB + C method (Yu and Morrison, 2004). Samples of concentrated bacterial cells were homogenized with zirconium beads and lysis buffer (1 mL), followed by centrifugation, and then the supernatant was recovered. Next, a mixture of 10 mM ammonium acetate, 98% isopropanol, and 70% ethanol was added to the supernatant for nucleic acid precipitation. Finally, the columns of the QIAamp DNA Mini Kit (QIAGEN, Hilden, Germany) were used for protein removal and DNA purification. The genomic DNA concentration was measured with a Qubit 3.0 fluorometer (Life Technologies, Carlsbad, CA, USA), and DNA integrity was verified by agarose gel electrophoresis (1%). The genomic DNA concentration obtained was 610 ng/mL with a 260/280 ratio of 1.86.

### Microbiota 16S rRNA Illumina Sequencing

The genomic DNA of the microbiota was used for 16S rRNA gene sequencing at Research and Testing Laboratory (RTL) Genomics (Lubbock, TX, USA) using the Illumina My-seq platform. Amplicon sequencing was performed for the V3-to-V4 region. Paired-end sequencing was carried out with a read length of 250 base pairs. The sequencing libraries were prepared using adapter regions compatible with the Nextera XT Index Kit (Illumina, San Diego, CA, USA). Data quality control and analyses were assessed using the USEARCH V.11 pipeline (https://drive5.com). The FASTQ forward and reverse files were merged into a single FASTQ file per sample. Quality control and processing included removing adapters and cutting the sequences to length-based filtering of 400 bp (reads smaller than 200 bp were excluded from the analysis). The filtered reads were then aligned with the RDP V.16 database to define operational taxonomic units (OTU) for taxonomy assignment, and the OTU table was generated at 97% identity. The Uclust method was used to cluster the reads into OTU (Edgar, 2010). R packages (Bioconductor, Phyloseq, Microbiome, Microbiomeutilites) were used to analyze microbiome data. The results of taxonomic composition are expressed as relative abundance (%) at the genus level.

### Animal Experiment

The protective effect of the DFM (A1D42) against enteric permeability was assessed in an in vivo model employing 18 clinically healthy male Sprague–Dawley rats of 3 wk of age and 47.1 ± 4.3 g of live body weight (Vuong et al., 2021). The animals were maintained at 24 ± 1 °C under a 12:12 (L:D) h photoperiod. The protocol was ethically approved by the Internal Committee for the Care and Use of Experimental Animals (SICUAE.MC-2021/4-4, UNAM). For the experiment, the rats were randomly divided into 3 experimental groups (*n* = 6): 2 received an oral gavage of DFM (0.5 mL) at doses of 10^9^ and 10^11^ CFU/mL, respectively, while the control group was administered with water (0.5 mL). Each group placed individual rats in cages as the experimental unit. The oral gavage administration was carried out daily for 2 wk. The probiotic doses were chosen based on preliminary experiments in our laboratory and according to the FAO/WHO recommendation to guarantee the concentration of at least 10^6^ viable CFU/mL (FAO/WHO, 2001).

Afterward, a single dose of 0.5 mL of FITC-d solution (8.32 mg/kg BW) was administered to evaluate intestinal permeability. The FITC-d solution was prepared 30 min before oral gavage administration. One-hour post-FITC-d administration, the animals were anesthetized with CO_2_ and sacrificed by guillotine decapitation. Immediately after decapitation, the blood of each animal was collected and centrifuged to obtain serum samples. These serum samples were protected from light and frozen at −70 °C. In addition, caeca and ileum samples and their digesta contents were collected. Samples were fixed in 4% paraformaldehyde (PFA) for histological analysis, while fecal contents were immediately frozen, placed in dry ice, and stored at −70 °C until further analysis.

### Fluorescein Isothiocyanate Dextran (FITC-d) Assay

Fluorescein isothiocyanate dextran (FITC-d, MW 3 to 5 kDa; Sigma Aldrich Co., St. Louis, MO, USA) was used as a mucosal barrier dysfunction marker, following the recommendations of Vuong et al. (2021). Briefly, 10 mg of FITC-d were reconstituted in 1 mL of 0.9% saline for the standard curve protocol, and the solution was protected from light. Next, the stock solution was used to construct a standard curve (1 to 32 μg/mL) in a 96-well black flat-bottom microplate. Fluorescence was measured in a Varioskan flash spectrophotometer (Thermo Fisher Scientific, Waltham, MA, USA) at 495 nm excitation and 519 nm of emission wavelengths, with a gain of 40. The results are expressed as microgram of FITC-d per mL of serum. Saline was also used as a diluent.

### Histological Analysis

Samples were dehydrated, embedded in paraffin, cut into 5-μm thick sections, and stained with the hematoxylin and eosin (H&E) protocol. Representative images were taken from each rat by group (*n* = 6). The images were captured at 10× and 40× magnification using a microscope (Leica, Microscopy, Germany) and analyzed with the ImageJ and Fiji software (Schindelin et al., 2012). The length and width of villus and crypts, the submucosal thickness, and Goblet cell counts were measured and averaged across 20 villi and 20 crypts per tissue section to 6 tissue sections per animal (Ribeiro et al., 2023).

### Experimental Design and Statistical Analysis

The physicochemical characterization was measured in triplicate, and results are expressed as mean ± standard deviation. Data from DFM rRNA 16S were analyzed and visualized using Rstudio software (http://www.rstudio.com) with the packages of the Phyloseq library (McMurdie and Holmes, 2013) and Bioconductor. In the in vivo study, normality and homoscedasticity tests were carried out using the Kolmogorov–Smirnov and Levene test, respectively. Mean values were subjected to analysis of variance (ANOVA), and comparative mean differences were determined by the Tukey test (*P* < 0.05). All statistics analyses were performed using Minitab Statistical Software V18 (Penn State University, State College, PA, USA). In addition, some graphics were generated with the GraphPad Prism software version 10 (San Diego, CA, USA).

## Results

### Physicochemical Characterization

#### Fourier-transform infrared spectroscopy.

**Figure 1.**
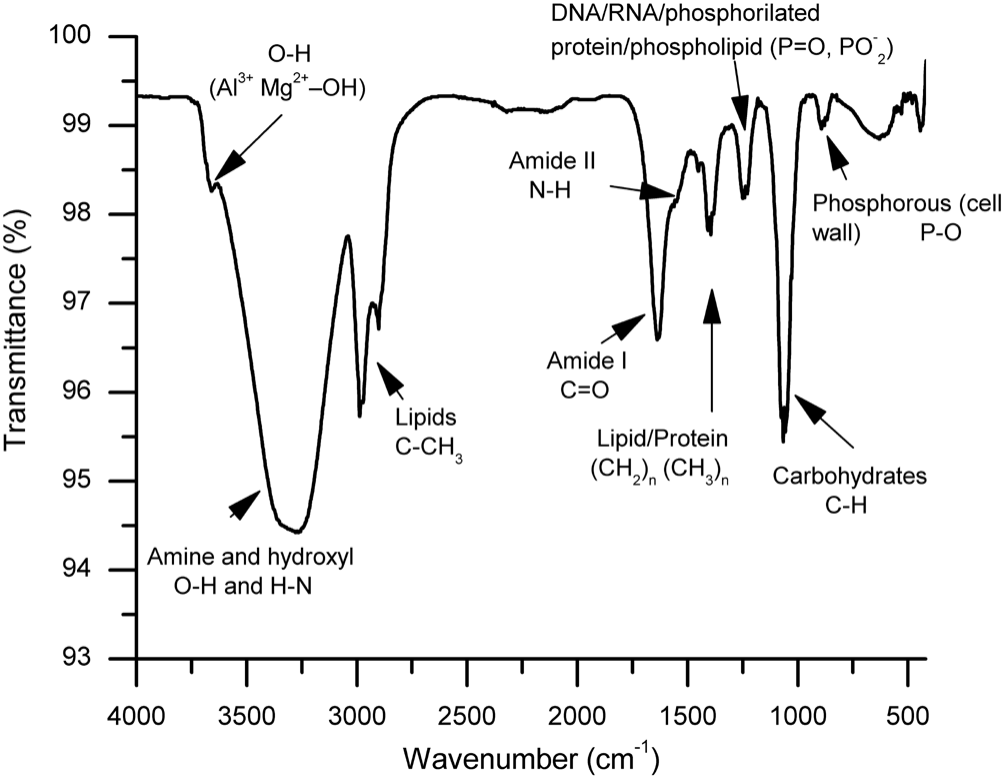
Representative Fourier-transform infrared (FTIR) spectra of the DFM.

In the spectrogram, the N–H asymmetric stretch of the amine and the O–H bond of the hydroxyl groups appeared at 3,270 cm^−1^, and alkyl chains appeared in the lipid region (between 3,000 and 2,800 cm^−1^). For instance, the bands at 2,987, 2,973, and 2,900 cm^−1^ represent the asymmetric CH_3_ stretching, the asymmetric CH_2_ stretching, and the symmetric CH_3_ stretching of membrane fatty acids (phospholipids), respectively. Moreover, the peptide-protein

 region occurred at frequencies between 1,650 and 1,500 cm^−1^. In this region, the carbonyl stretching of amide I and the N–H bending of amide II were located at 1,637 and 1,548 cm^−1^, respectively. Furthermore, in the FTIR spectrum, the band located at 1,395 cm^−1^ is associated with the –(CH_2_)_*n*_ vibration of lipid and protein, and the band centered at 1,241 cm^−1^ is attributed to the P=O vibration of DNA, RNA, phospholipid, and phosphorylated protein. The molecular vibration of saccharides and phosphate appeared in the range of 1,074 to 1,057 cm^−1^, and the band at 893 cm^−1^ corresponded to the P–O vibration from phosphorus present in the cell wall (Fig. 1). In general, it was observed that the A1D42-DFM had a protein/carbohydrate ratio of 1.3:1 based on its computed regions bands ratio.

#### Hydrodynamic size and PDI.

**Figure 2.**
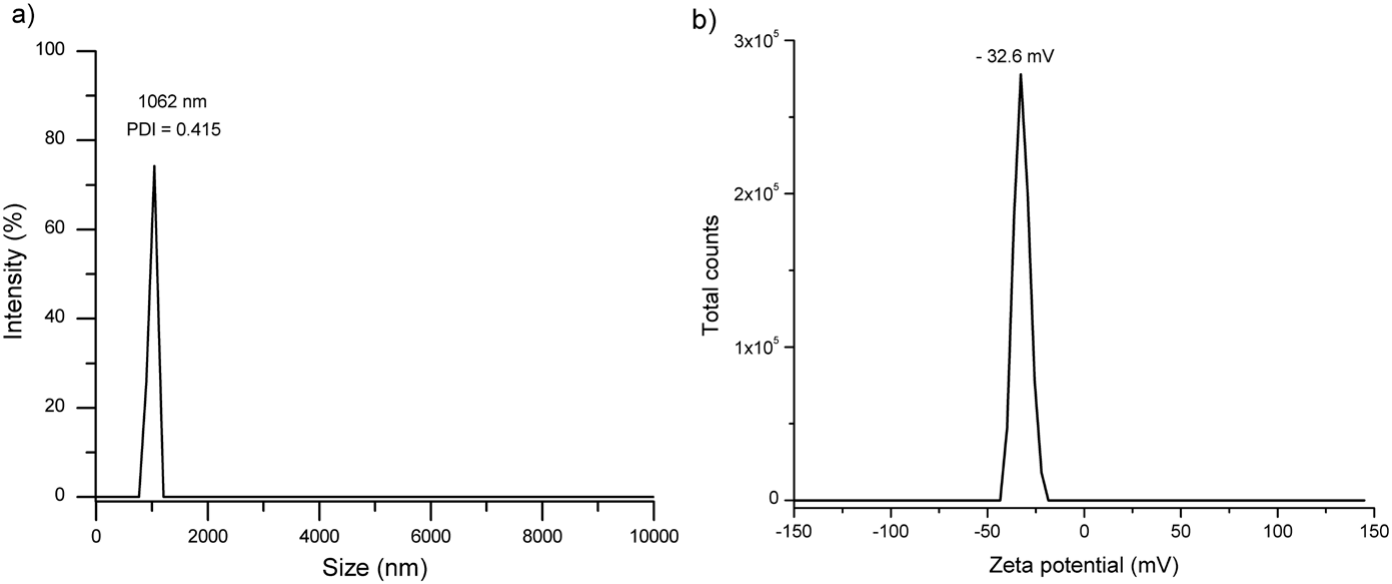
Hydrodynamic diameter and PDI (A) and zeta potential (B) of the DFM.

The particle size distribution (in intensity percentage) was relatively narrow, with an average size of 1,062 ± 77 nm. Moreover, the DFM yielded a PDI of 0.415, indicative of a nearly monodisperse sample (Fig. 2A).

#### Zeta potential.

*Using* the electrophoretic mobility technique, the zeta-potential value attained was −32.6 ± 3.7 mV, indicative of a highly electronegative bacterial cell surface (Fig. 2B).

#### Cell surface hydrophobicity.

The DFM showed a high hydrophobicity value (92%), suggesting the sample had a rich protein surface.

#### ESEM and EDS analyses.

**Figure 3.**
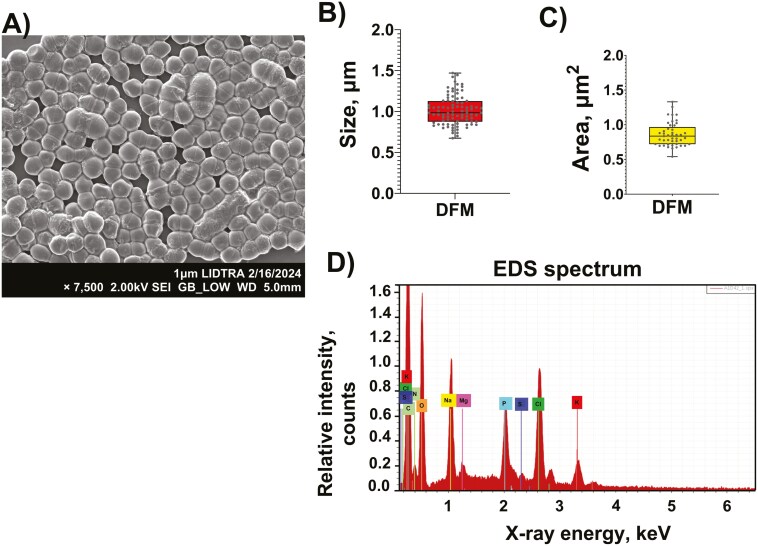
ESEM image (A), size (B), area (C), and EDS spectra (D) of the DFM.

In general, the micrograph showed the characteristic shape of the bacterial cells (cocci-shaped or quasi-spherical). Additionally, the A1D42-DFM mainly contains isolated cells and minor aggregate/agglomerate ones with a smooth surface microstructure. Moreover, no significant alterations in terms of cell membrane integrity were noticed (Fig. 3A). Furthermore, cells appear quite regular in shape, showing a narrow variability, as confirmed by the measurement option of their size and area distribution (Fig. 3B and C). The average size and area of the half population were around 1,010 ± 180 nm and 890 ± 170 nm^2^, respectively.

On the other hand, the micro-elemental analysis of the DFM was accomplished using the energy-dispersive X-ray fluorescence technique. The micro-elemental analysis revealed the presence of significant amounts of carbon (54.42%) and oxygen (28.10%). Moreover, considerable intensities of nitrogen (10.14%), sodium (3.02%), chlorine (2.16%), and phosphorus (1.22%) were also found. Finally, low contents of potassium (0.62%), magnesium (0.26%), and sulfur (0.07%) were also detected (Table 1). With this information, the nitrogen over carbon (N/C) ratio was calculated to measure proteinaceous groups on the cell surface. Additionally, the DFM showed a high N/C surface ratio (0.186), indicating considerable amounts of surface layer proteins.

**Table 1.** The elemental composition (%) of the DFM determined by energy-dispersive X-ray spectroscopy

Elemental	Atomic (%)	Weight (%)
C	54.42 ± 0.40	44.72 ± 0.32
N	10.14 ± 0.37	9.72 ± 0.35
O	28.10 ± 0.29	30.76 ± 0.31
Na	3.02 ± 0.08	4.75 ± 0.12
Mg	0.26 ± 0.02	0.42 ± 0.04
P	1.22 ± 0.02	2.57 ± 0.05
S	0.07 ± 0.03	0.16 ± 0.06
Cl	2.16 ± 0.02	5.23 ± 0.06
K	0.62 ± 0.03	1.66 ± 0.08

The results are expressed as mean ± standard deviation (*n* = 3).

### 16S rRNA Sequencing

**Figure 4.**
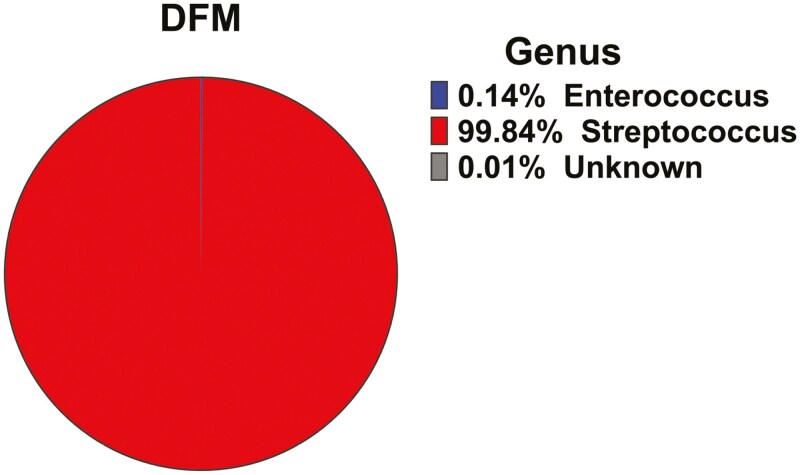
The relative abundance (%) of bacteria of the DFM examined at the level of the genus based on the quality sequences.

Figure 4 shows the relative abundance at the genus level of the DFM (A1D42) analyzed by the 16S rRNA sequencing. *Streptococcus* was the most predominant bacterial population (99.84%), followed by *Enterococcus* (0.14%). Less than 0.02% of the bacterial population was not classified through the USEARCH pipeline.

### In Vivo Experiment

**Figure 5.**
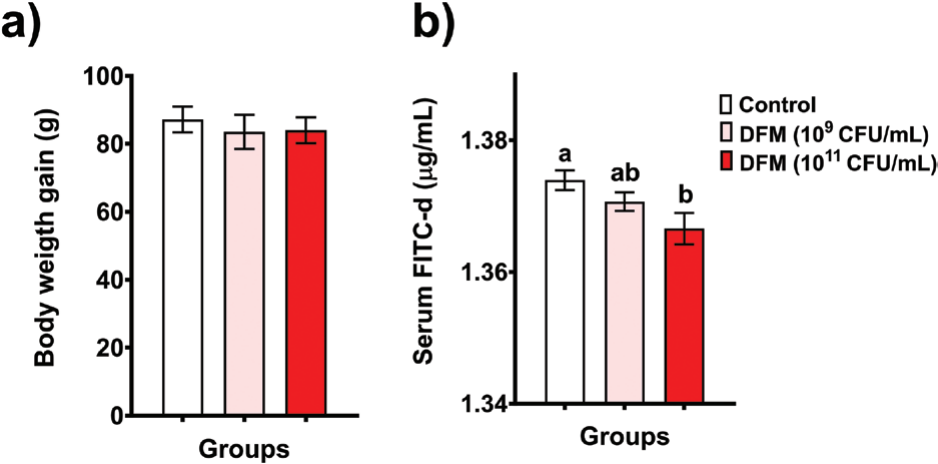
Body weight gain (A) and serum FITC-d concentration (B) of Sprague-Dawley rats supplemented with 2 doses of the DFM. Means values not sharing a common superscript letter(s) are significantly different (*P* < 0.05).

In general, both concentrations (10^9^ and 10^11^ CFU/mL) of the DFM tested did not cause visible health damage, diarrhea, or other complications in the rats throughout the 2 experimental weeks. Moreover, no significant changes were observed in the body weight gain between the control and treated groups (Fig. 5A). Figure 5B shows the serum concentrations of FITC-d. The animals supplemented with the higher dose of DFM (10^11^ CFU/mL) significantly reduced (*P* < 0.05) the intestinal permeability of the marker compared to the control group. Although the low dose of the DFM could also reduce the serum concentration of FITC-d (numerically), it was not statistically significant.

**Figure 6.**
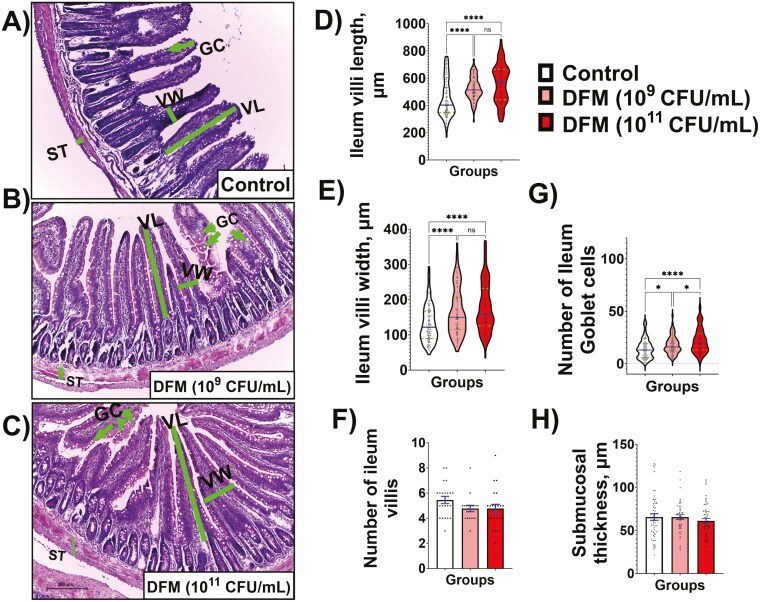
Representative H&E ileum histology (10× magnification, scale bar = 200 µm) of control (A) and supplemented groups with 2 doses of DFM (B and C). The lines indicate dimensions measured for villi length (VL), villi width (VW), and submucosal thickness (ST), while arrows indicate Goblet cells. Quantitative comparison regarding villus length (D), villus width (E), number of villus (F) Goblet cells (G), and submucosal thickness (H) measured per each experimental group. Approximately 20 villi and their Goblet cells spanning the length of the ileal intestine were measured per animal. Data are expressed as means ± SE. Statistical differences were determined by 1-way ANOVA and Tukey tests and denoted as follows: * for *P* < 0.05, ** for *P* < 0.01, *** for *P* < 0.001, and ns for nonsignificant differences.

Furthermore, the effects of the DFM supplementation on ileal tissue structure are shown in Fig. 6. Histological images revealed typical small intestine architectures in the control group (Fig. 6A). Interestingly, a larger dimension of the villi and a more significant number of Goblet cells were observed in the ileal intestine micrographics of rats supplemented with both doses of the DFM (Fig. 6B and C, respectively). Thus, rats supplemented with both concentrations of the DFM showed significantly greater length and width of ileal villi than the control group (Fig. 6D and E, respectively). In addition, both DFM doses significantly increased the number of Goblet cells compared with the control group. Overall, the supplementation with the DFM at the higher dose (10^11^ CFU/mL) exerted the most significant changes on increased Goblet cells (Fig. 6F). Finally, no statistical differences were observed in the number of villi and the submucosal thickness between the 3 experimental groups (Fig. 6G and H, respectively).

**Figure 7.**
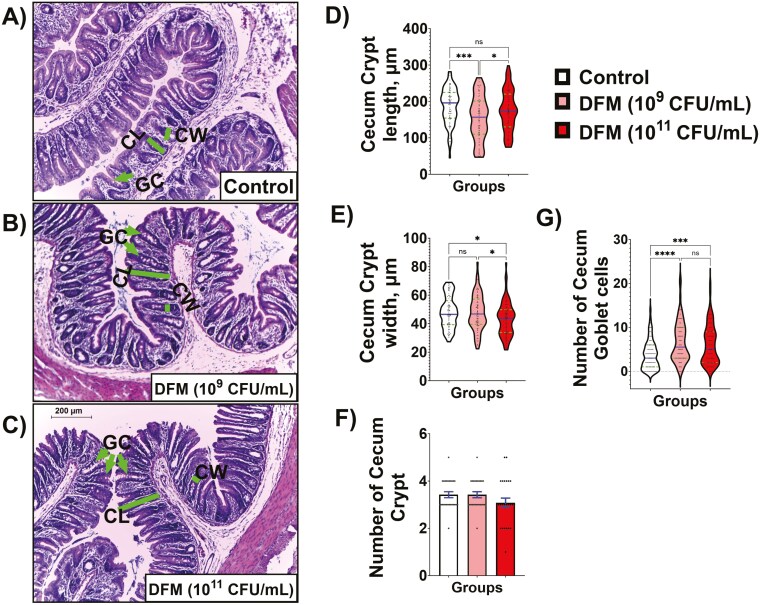
Representative H&E cecum histology (10× magnification, scale bar = 200 µm) of control (A) and supplemented groups with 2 doses of DFM (B and C). The lines indicate dimensions measured for crypt length (CL) and width (CW), while arrows indicate Goblet cells. Quantitative comparison regarding crypt length (D), crypt width (E), the number of crypts (F), and Goblet cells (G) measured per each experimental group. Approximately 20 crypts and their Goblet cells spanning the length of the caeca intestine were measured per animal. Data are expressed as means ± SE. Statistical differences were determined by 1-way ANOVA and Tukey tests and denoted as follows: * for *P* < 0.05, ** for *P* < 0.01, *** for *P* < 0.001, and ns for nonsignificant differences.

Additionally, the histological architecture of the cecum region was analyzed. Representative micrographics and their results are shown in Fig. 7. Typical caeca morphologies were found in the tissues of the control group (Fig. 7A). Histological analysis of the rats supplemented with DFM revealed no apparent changes in cecum crypt dimensions (Fig. 7B and C, respectively). Semi-quantitative image analysis also showed no statistical differences in crypt length at higher doses compared to the control group. However, a significant decrease in crypt length was observed at lower DFM doses (Fig. 7D). Regarding crypt width, no significant changes were detected at lower DFM doses, whereas a decrease was observed at higher doses (Fig. 7E). Besides, the DFM-supplemented groups showed a higher number of Goblet cells than the control group (Fig. 7F), and no significant differences in the number of crypts per animal across the groups were observed (Fig. 7G).

## Discussion

In this study, the physiochemical and metagenomic analysis of a DFM isolated from ruminal fluid of calves was determined and its potential effect on intestinal permeability was also evaluated. This study is a trailblazer utilizing FTIR, DLS, and ESEM-EDS techniques for physicochemical characterization. The findings obtained with these techniques were corroborated by the metagenomic 16S analysis, highlighting their potential application in animal livestock.

The FITR technique has been successfully used in investigating bacterial strains in the clinical field and recently in probiotics for the food industry (Wenning et al., 2010; Dziuba and Nalepa, 2012; Deidda et al., 2022). The FTIR fingerprint represented an overview of chemical constitutions in terms of the functional groups of the bacterial cells, which can be inferred from feasible and reliable identification procedures (Wenning et al., 2010; Deidda et al., 2022). In this context, Naumann et al. (1991) mentioned 5 IR spectral regions that can be distinguished for bacterial identification objectives: the fatty acid region (3,000 to 2,800 cm^−1^), the amide I and II bands of proteins and peptides (1,700 to 1,500 cm^−1^), mixing bending vibrational regions of fatty acids, proteins, and molecules with phosphates-carryings (1,500 to 1,200 cm^−1^), bands of carbohydrate region (1,200 to 900 cm^−1^), and the fingerprint bands of the specific bacteria (900 to 700 cm^−1^). In line with these results, the FTIR spectra of the A1D42-DFM showed the leading characteristic bands of these 5 spectral regions of probiotic cells. In this work, the ratio of areas corresponding to the protein and carbohydrate bands was computed as a useful indicator of key surface components. Consequently, the FTIR technique confirmed that the DFM had a relatively high protein-rich surface. For example, Wenning et al. (2010) demonstrated the effectiveness of FTIR spectroscopy in identifying 92 lactic acid bacteria, including *Lactobacillus*, *Lactococcus*, and *Streptococcus*, among others. The authors reported an adequate classification rate of 92% for these bacterial strains and concluded that this method might enable routine laboratories to identify them. Another study also analyzed these genera of bacteria using FTIR spectroscopy in 2 stages. The first stage used reference strains, while the second focused on bacteria isolated from food products. This approach resulted in a 95% correct identification of lactic acid bacteria (Dziuba and Nalepa, 2012). In the present study, we focused on characterizing the A1D42-DFM strains isolated from the ruminal fluid of calves. According to the FTIR results, it may be hypothesized that they might belong to the *Lactobacillus* or *Streptococcus* genera; however, at this experiment stage, it was still necessary to complement and corroborate these findings with other physicochemical and metagenomic methods.

One of the methods to assess the average diameter of particles in suspension is by using DLS. In this research, the DFM average size was 1,062 ± 77 nm, and particles appeared monodisperse based on the PDI value. This particle size value provides a representative average of the bacterial cell population. Moreover, the curve provides a qualitative indication of the dispersion in terms of homogeneity. In general, size and PDI results are in close agreement with those of bacterial cells measured by the DLS technique (Razafindralambo et al., 2019).

The zeta-potential results clearly reflect the surface chemical composition of the bacterial cells in the A1D42 consortium (DFM), which, in turn, depends on the ionized or non-ionized state of certain chemical functional groups. This electronegative potential (−32.6 ± 3.7 mV) is mainly attributed to anionic domains of various surface compounds present on the cell wall, such as phosphate-based (lipo-) teichoic acids and carboxylate-containing acidic polysaccharides and proteins (Schär-Zammaretti et al., 2005). This result was well corroborated by the FTIR measurements since the DFM had a protein-rich surface. In the context of the surface charge, Van Der Mei et al. (2003) reported a zeta-potential value of approximately −30 mV for a *Lactobacillus casei* 393*/CA5ʹA strain at neutral pH.

On the other hand, bacterial adhesion to hydrocarbons is a simple and rapid technique for evaluating cell surface hydrophobicity. Hydrophobicity is one of the main physical interactions during bacterial adhesion to epithelial cells (Canzi et al., 2005). In this research, the methodology was performed at pH 3 because, at this acidic pH, hexadecane should not be charged; consequently, electrostatic interactions are negligible (Pérez et al., 1998). The high hydrophobicity of the DFM (92%) is consistent with previous reports with probiotic strains such as *Lactobacillus* (El Hage et al., 2022) and *Streptococcus* (Chen et al., 2020). This observation is also in close agreement with the FTIR results, which indicated that the DFM had a protein-rich surface. Thus, proteins on the cell´s surface largely contributed to the hydrophobic character of the DFM cells.

Several works have also shown that the hydrophobicity of *Lactobacillus* and other bacteria-produced lactic acid is mainly due to a protein-rich surface. For instance, Deepika et al. (2009), using *L. rhamnosus* GG (ATCC 53103), demonstrated that adhesion to hexadecane significantly increased with growth time, reaching a maximum at 13 h (up to 85%); however, the SDS-PAGE analysis showed the lowest content of surface proteins. The authors conclude that proteins found in these cells were highly hydrophobic. Furthermore, Espeche et al. (2009) screened the surface properties of lactic acid bacteria, including *Lactobacillus* and *Streptococcus* strains. In general, *Streptococcus* exhibited a hydrophobicity surface with an auto aggregation phenotype towards hexadecane. The authors suggested that this phenotype could be used to predict the high possibilities of mucus adhesion and the formation of a protective biofilm once administrated to the host. In our previous work (Rodríguez-González et al., 2022a), we demonstrated the in vitro adhesion properties of the DFM to ruminant intestinal mucus. The DFM showed a significant ability to colonize the mucus obtained from calves, indicating potential benefits for protecting intestinal integrity.

The ESEM-EDS results showed that the DFM had a quasi-spherical or cocci-like morphology, with sizes ranging up to 1,010 nm. Regarding the micro-elemental analysis, the N/C surface ratio of the DFM is comparable with the N/C ratios of 0.172 and 0.160, measured for *L. acidophilus ATCC4356* and *L. crispatus JCM5810,* respectively (Van Der Mei et al., 2003). The rRNA 16S sequencing analysis revealed that *Streptococcus* was the predominant genus in the DFM, with a relative abundance of 99%. This result was well corroborated by the FTIR fingerprint, zeta potential, and cell surface hydrophobicity measurements. Additionally, the physicochemical characterization of various *Streptococcus* strains that have been previously evaluated is in agreement with the results of this experiment (Espeche et al., 2009). Several species of *Streptococcus* are known to be responsible for numerous animal diseases. However, many others are nonpathogenic and occur as natural commensal microbiota. Several *Streptococcus* strains are considered by the Food and Agriculture Organization of the United States as probiotics used in animal diets (Bajagai et al., 2016).

Once the DFM was further characterized, it was evaluated against intestinal permeability in an in vivo experiment. One of our primary considerations was to ensure the safety of the DFM by assessing its potential association with infections, induction of diarrhea, or toxicity. Our experimental model showed no adverse effects or illnesses in healthy rats after 2 wk of DFM administration using 2 doses, which could support their safety. Furthermore, the DFM exhibited the ability to reduce the intestinal permeability of FITC-d in the experimental rats. In addition, the length and width of villi and crypts and the number of Goblet cells increased in both the ileal intestines of rats supplemented with the DFM.

Intestinal permeability has been associated with changes in villus and crypt structure in the small and large intestines, including changes in the number of Goblet cells and their mucus secretion and function (Ribeiro et al., 2023). The height of the villi and the length of the crypts are indicators of intestinal function. Consistent with other studies, the administration of the DFM improved the villus and crypt structures, increasing their dimension with a higher number of Goblet cells (Krehbiel et al., 2003; Dick et al., 2013). The DFM can act by different mechanisms. Supplementation of DFMs can modulate the host’s immune response, reducing gut inflammation and promoting the repair of damaged epithelial cells (Krehbiel et al., 2003). Also, DFMs can produce various metabolites, such as short-chain fatty acids, which serve as an energy source for the epithelial cells and help maintain their integrity (Wu et al., 2022). It has been reported that supplementation of DFM in neonatal calves improves diarrhea, intestinal immune function, and inflammation by regulating the intestinal microbiota (Abd El-Tawab et al., 2016). As aforementioned, the DFM can also enhance the population of beneficial bacteria by competitive exclusion of pathogenic bacteria. This helps prevent colonization and reduce the risk of epithelial damage. These effects contribute to maintaining a healthy intestinal epithelium with longer villi and deeper crypts, enhancing nutrient absorption and overall gut health.

These findings highlight the important properties of DFM for further experiments in ruminants, especially their safety and gut health benefits. Given these promising results, DFM could potentially enhance gut health and improve overall ruminant performance. However, it is essential to conduct long-term in vivo studies to assess the full spectrum benefits of DFM and potential limitations in ruminants, including any species-specific responses and environmental factors that could influence its efficacy. Moreover, the point of view of differences in the gastrointestinal physiology between rats and ruminants should be carefully considered.

On the other hand, FTIR, DLS, and MATH assay are cost-effective and efficient techniques for characterizing DFMs, offering valuable insights into biochemical composition, particle size distribution, surface charge, and bacterial hydrophobicity. However, their widespread adoption is hindered by limitations such as the complexity of interpreting FTIR results without adequate expertise or comprehensive databases, the inability of DLS to analyze complex microbial consortia, and the low sensitivity of the MATH assay, which requires validation with advanced methods. Additionally, the lack of standardized protocols, awareness, and training among researchers and industries further restricts their application. Consequently, molecular techniques like 16S rRNA sequencing and metagenomics remain the gold standard due to their higher taxonomic resolution. Therefore, future research should focus on expanding databases and promoting these techniques as complementary tools for probiotic characterization.

## Conclusions

The FTIR, DLS, and bacterial cell surface hydrophobicity are effective and low-cost physicochemical techniques to identify the main characteristics of the *Streptococcus*-based DFM strain. These findings were well corroborated by the 16 rRNA sequencing and its morphology and micro-elemental composition by ESEM-EDS. Thus, these methods could be applied in the probiotics field for the livestock industry. Furthermore, the DFM supplementation exhibited nonpathogenic and nontoxic effects in healthy rats and improved the intestinal barrier function by increasing the villi and crypt length in small and large intestines with more Goblet cells. These results support the potential benefits of DFM supplementation in ruminants to enhance performance and qualify to be included in the list of “generally recognized as safe” substances.

## Data Availability

The Raw data reported in this study can be found in BioProject RJNAF945191 of the Sequence Read Archive (SRA) database of the NCBI.
